# Thermal Degradation Kinetics and Viscoelastic Behavior of Poly(Methyl Methacrylate)/Organomodified Montmorillonite Nanocomposites Prepared via In Situ Bulk Radical Polymerization

**DOI:** 10.3390/polym10050491

**Published:** 2018-05-03

**Authors:** Alexandros K. Nikolaidis, Dimitris S. Achilias

**Affiliations:** Laboratory of Polymer and Colour Chemistry and Technology, Department of Chemistry, Aristotle University of Thessaloniki, 541 24 Thessaloniki, Greece; nikolchem@dent.auth.gr

**Keywords:** nanocomposites, PMMA, montmorillonite, thermal degradation kinetics, viscoelastic properties

## Abstract

Nanocomposites of polymers with nanoclays have recently found great research interest due to their enhanced thermal and mechanical properties. Deep understanding of the kinetics of thermal degradation of such materials is very important, since the degradation mechanism usually changes in the presence of the nano-filler. In this investigation, poly(methyl methacrylate)/organomodified clay nanocomposite materials were prepared by the in situ free radical bulk polymerization technique. The thermal degradation of the products obtained was studied by means of thermogravimetric analysis at several heating rates. Isoconversional kinetic analysis was conducted in order to investigate the effect of degradation conversion on the activation energy. Both, pure poly(methyl methacrylate) (PMMA) and its nanocomposites were found to degrade through a two-step reaction mechanism. Data arising from a differential and an integral method were used to disclose the correlation between activation energies (*E_α_*) and the extent of degradation (α). It was found that *E_α_* value improved for all nanocomposites at α values higher than 0.3. Moreover, the viscoelastic behavior of the obtained nanocomposites was examined by means of dynamic mechanical thermal analysis. All nanocomposites exhibited higher storage modulus in comparison to the virgin PMMA at room temperature, while the increment of clay amount improved their stiffness gradually.

## 1. Introduction

In recent years, the research attention has been focused on polymer/clay nanocomposites which consist of polymer matrices containing platelet-shaped clay particles that have sizes on the order of a few nanometers in thickness and several hundred nanometers in length [1]. It has been observed that the presence of layered silicate materials, such as montmorillonite (MMT), hectorite, bentonite, and so on, at relatively low loading levels (typically 3–5 wt %) can greatly enhance barrier properties and improve the mechanical properties, thermal stability and fire retardancy of polymers [2,3,4,5,6,7,8,9]. The significant advantage in the formation of nano-composites compared to micro-composites is that the polymeric chains intercalate between the silicate sheets, resulting in the formation of a uniform material instead of a phase-separated composite. Two types of nanocomposites can be recovered, namely intercalated and exfoliated. In the first structure, macromolecular chains are intercalated between the silicate layers resulting in a well ordered multilayer morphology built up with alternating polymeric and inorganic layers. In the latter structure, the silicate layers are completely and uniformly dispersed in a continuous polymer matrix [2]. The methods proposed for producing polymer/layered silicate nanocomposites can be classified as: (1) in situ template synthesis; (2) solution casting; (3) melt intercalation; and (4) in situ polymerization [2,10,11,12,13,14]. Among them, the in situ polymerization seems to be the most desirable and effective method for the production of nanocomposites, since it usually leads to uniform materials [15]. The compatibility between clay and organic matrix is also a critical point for the high fidelity production of polymer/clay nanocomposites [16].

Thermal stability comprises a crucial parameter for the production and the specific application of a polymer/clay nanocomposite. The kinetics of polymer thermal degradation has been extensively studied in literature using mainly Thermogravimetric analysis (TGA). In this context the mass loss of a material is continuously recorded as a function of increasing temperature at several heating rates. In order to provide an insight into the thermal degradation mechanism, model-fitting, or model-free (i.e., isoconversional) methods are usually employed [17,18,19,20,21,22,23,24,25,26,27]. A brief review of the literature on the thermal degradation kinetics of polymer/clay nanocomposites follows. Kandare et al. [20] calculated the activation energy, *E_α_*, of thermal decomposition for poly(methyl methacrylate) (PMMA)/clay composites as a function of conversion. Analysis of multiple heating rate data using the Flynn–Wall–Ozawa (FWO) method resulted in *E_α_* values that in the composite materials were 50 kJ/mol higher compared to neat PMMA [20]. Erceg and coworkers [19] conducted kinetic analysis of the non-isothermal degradation of poly(ethylene oxide) (PEO)/organically modified MMT (OMMT) nanocomposites, using the isoconversional FWO and Friedman (FR) methods to calculate the kinetic parameters of the process. They found that the activation energies, *E_FWO_* and *E_FR_* did not practically depend on the extent of degradation for all samples. From a kinetic point of view this means that the non-isothermal degradation of PEO and PEO/MMT nanocomposites was a one-step process. Both E*_FWO_* and E*_FR_* reached their maximum mean value (in the conversion range *α* = 0.1–0.9) for nanocomposites containing 1 wt % MMT [19]. Polystyrene (PS)/clay nanocomposites were studied by Bourbigot et al. [25] in terms of their kinetics of thermal and thermo-oxidative degradation on the basis of a model-free Friedman analysis. Graphs of the activation energy revealed that a multi-step degradation mechanism occurs. In addition, thermal degradation of pristine PS exhibited a two reaction mechanism, whereas a four-reaction model was proposed for the thermo-oxidative degradation of its nanocomposites [25]. Vyazovkin et al. [26,27] also studied the thermal and thermo-oxidative degradation of PS and PS/clay nanocomposites. The advanced isoconversional method, developed by the same prime author, was applied for kinetic analysis. They found that introduction of the clay in the polymer matrix, increased the activation energy and affected the total heat of degradation. This is an indication of a change in the reaction mechanism [17,26,27]. The obtained kinetic data permitted comparative assessment of fire resistance of the studied materials [26,27]. Although the thermal stability of PMMA/clay nanocomposites has been investigated in literature [28,29,30,31,32,33,34,35,36,37], a detailed kinetic analysis of thermal decomposition for PMMA/MMT nanocomposites has not been systematically proposed so far.

In this study, the effect of the type of organic modifier of OMMT nanoparticles and their amount on the thermal degradation kinetics of PMMA/MMT nanocomposites is investigated. Thermogravimetric (TG) experiments at different heating rates were performed and isoconversional analysis, using a differential and an integral method was carried out in order to determine the alteration of the effective activation energy as a function of the extent of degradation. In addition, viscoelastic performance of the above nanocomposites using DMTA was investigated in order to estimate the effect of the nano-filler on the elastic and loss moduli of the materials prepared.

## 2. Materials and Methods

### 2.1. Materials

The monomer used was methyl methacrylate (MMA) (Fluka, purity ≥ 99%). Hydroquinone inhibitor was removed by passing it, at least twice before any use, through a disposable inhibitor-remover packed column (from Aldrich, St. Louis, MO, USA). Benzoyl peroxide (BPO) (Fluka, purity > 97%) was used as a radical initiator, purified by fractional recrystallization twice from methanol (Merck, Kenilworth, NJ, USA). All other chemicals used were of reagent grade.

The clays used were commercial OMMT under the trade names Cloisite 15A, Cloisite 25A and Cloisite 30B, (Southern Clay Products Inc., Gonzales, TX, USA), modified with a quaternary ammonium salt, which was dimethyl hydrogenated tallow, dimethyl 2-ethyl hexyl hydrogenated tallow and methyl tallow bis-2-hydroxyethyl, respectively. OMMT clay, as prepared in our previous work with cetyltrimethylammonium cations (AA-MMT) [38], was also used for this purpose.

### 2.2. Preparation of the Initial Monomer-Clay Mixture

The appropriate amount of OMMT was dispersed in 25 g of MMA, monomer in a 100 mL conical flask, by adequate magnetic and ultrasound agitation. The duration of agitation was varied depending on the type of OMMT used. Thus, the magnetic agitation lasted for 2, 15, 22.5 and 24 h, for Cloisite 15A, Cloisite 25A, Cloisite 30B and AA-MMT respectively, whereas the ultrasound agitation was 1 h, the same for all the samples. The dispersion of the OMMT in the monomer was homogeneous, as indicated by the high translucency in the visible region. Finally, the initiator, BPO was added at concentration 0.03 mol/L, same for all the experiments. The mixture was degassed by passing high purity nitrogen and immediately used.

### 2.3. Synthesis of PMMA/Clay Nanocomposites

The in situ bulk free radical polymerization technique was employed to prepare all PMMA/clay nanocomposite materials. A series of experiments was carried out keeping the amount of clay constant at 1 wt % and using Cloisite 15A, Cloisite 25A, Cloisite 30B and AA-MMT, whereas in another set of experiments Cloisite 15A was added at different initial amounts ranging from 1 to 5 wt %. Neat PMMA was also synthesized. According to the experimental procedure, each mixture was initially heated to 80 °C for a suitable time period, to get a critical solution viscosity. Then, the viscous liquid obtained was poured into a mould to complete the polymerization process successfully at 40 °C for 20 h and prepare appropriate specimens for the thermal and mechanical property measurements.

### 2.4. Measurements

*Transmission Electron Microscopy* (*TEM*). For the TEM measurements, a JEOL 2011 TEM with a LaB6 filament and an accelerating voltage of 200 kV was used. In order to prepare the samples, drops of each PMMA/OMMT nanocomposite—ethanol suspension were evaporated after sonication onto a carbon-coated lacy film supported on a 3 mm diameter, 300 mesh copper grid.

*Thermogravimetric Analysis (TGA)*. To study the thermal degradation characteristics of the nanocomposites, TGA was carried out using a Pyris 1 TGA (Perkin Elmer, Waltham, MA, USA) thermal analyzer. Samples weighed approximately 5–8 mg and placed into a sample pan made of Pt. Heating was applied from ambient temperature to 600 °C under a 20 mL/min nitrogen flow. Four different heating rates were employed, that is, 2.5, 5, 10, and 20 °C/min, in order to have adequate data to perform kinetic analysis of the process. The variation of the sample mass with temperature was continuously recorded using a high precision balance.

*Dynamic Mechanical Thermal Analysis (DMTA)*. In order to perform dynamic thermo-mechanical analysis on the samples and study their viscoelastic behaviour, a Perkin Elmer Diamond DMTA (Technology SII) in sinusoidal three-point bending mode was employed. The vibration frequency was 1Hz, the stress at 4000 mN and the amplitude 10 mμ. The temperature heating program was varied from 25 to 200 °C with a scanning rate of 3 °C/min. Rod-like specimens were used having dimensions 2 × 2 × 40 mm.

## 3. Background Theory for the Kinetic Analysis of TG Data According to Isoconversional Methods

It is common practice to use the following simple equation for the description of the kinetics of polymer degradation [39,40,41]:

(1) $$\frac{d\alpha }{dt}=k(T)f(a)$$

with *a* representing the extent of reaction, determined from TG runs as a fractional mass loss and ranging from 0 to 1, *t* expressing time, *k(T)* a temperature-dependent kinetic rate constant and *f(a)* denoting the particular reaction model, which describes the dependence of the reaction rate on the extent of reaction. Using an Arrhenius-type expression for the temperature dependence of *k(T)*, Equation (1) is transformed to:

(2) $$\frac{d\alpha }{dt}=A\exp\left(\frac{-E}{RT}\right)f(a)$$

with *A* and *E* being the pre-exponential factor and the activation energy, respectively.

According to the isoconversional principle, at a constant extent of reaction, the reaction rate is a function only of the temperature. Hence Equation (2) can be written as:

(3) $${\left[\frac{d\ln\left(da/dt\right)}{d(1/T)}\right]}_{\alpha }=-\frac{{Ε}_{\alpha }}{R}$$

where the subscript *α* denotes the value at a specific extent of reaction.

Isoconversional methods provide a variation of *E_α_* with *α* usually employing multiple heating rates to obtain data on varying rates at a constant extent of conversion [40]. Taking the logarithm of Equations (2) and (4) can be obtained.

(4) $$\ln{\left(\frac{da}{dt}\right)}_{\alpha ,i}=\ln\left[A_{\alpha }f(\alpha )\right]-\frac{{Ε}_{\alpha }}{R{Τ}_{\alpha ,i}}$$

the subscript *i* denotes different heating rates.

Equation (4) constitutes the differential isoconversional method of Friedman [42], where the activation energy can be obtained from the slope of the straight lines obtained after plotting the left-hand side vs. 1/*T*. Since TG measurements provide data on the variation of the extent of conversion with temperature, in order to use Friedman’s method, numerical differentiation of the experimental curves is required. This is typically carried out by the software of the instrument used, and sometimes, depending on the quality of the measurements, it results in quite noisy rate data, and thus unstable activation energy values. The problem of numerical differentiation could be avoided by adopting integral isoconversional methods. For nonisothermal conditions, when the temperature is raised at constant heating rate *β*, integration of Equation (2) involves solving the temperature integral in Equation (5):

(5) $$g(a)\equiv \int_{0}^{\alpha }\frac{da}{f(\alpha )}=\frac{Α}{\beta }\int_{T_{0}}^{T_{\alpha }}\exp\left(\frac{-E}{RT}\right)dT=\frac{Α}{\beta }I(E,T)$$

However, the situation in Equation (5) is that the integral *I(E,T)* does not have an analytical solution. Therefore, either approximations or numerical integration are used to solve it. One of the simpler approximations was proposed by Doyle and results to the following Equation (6). This equation constitutes the popular isoconversional method of Flynn and Wall [43] and Ozawa [44] (FWO).

(6) $$\ln(\beta_{i}^{})=const.-\frac{1.05{Ε}_{\alpha }}{{RT}_{\alpha ,i}}$$

The use of a more precise approximation by Coats and Redfern [45] yields the following equation, usually known as KAS method after Kissinger-Akahira and Sunose:

(7) $$\ln\left(\frac{\beta_{i}}{T_{\alpha ,i}^{2}}\right)=const.-\frac{{Ε}_{\alpha }}{R{Τ}_{\alpha ,i}}$$

Usually, application of differential or integral methods result in systematic differences in the activation energies evaluated. As was reported previously, the differential method of Friedman employs instantaneous rate values, and, therefore, is susceptible to experimental noise, whereas in the integral methods, the equation used are derived assuming constant activation energy, introducing thus a systematic error in the estimation of *E*. The latter is large in the case that multiple reactions take place and *E* varies with *α* [39]. Further increase in precision of the integral methods can be accomplished by using numerical integration, a method developed and extensively used by Vyazovkin [46,47,48]. In this investigation the methods of Friedman, and Coats-Redfern were used.

## 4. Results and Discussion

### 4.1. Structure and Morphology of the Nanocomposites

**Figure 1:**
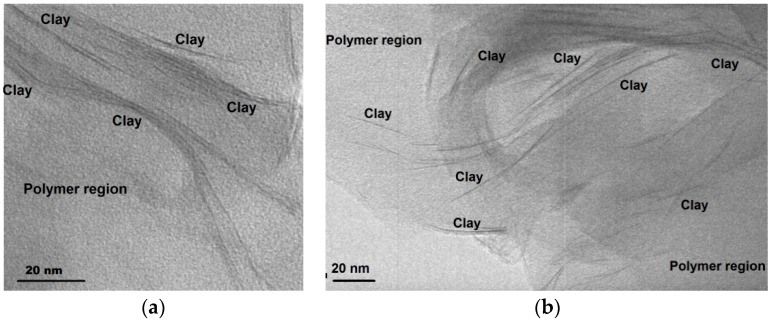
Transmission Electron Microscopy (TEM) images of poly(methyl methacrylate) (PMMA) nanocomposites containing 1 wt % Cloisite 15A (**a**) and 2 wt % Cloisite 15A (**b**).

**Scheme 1:**
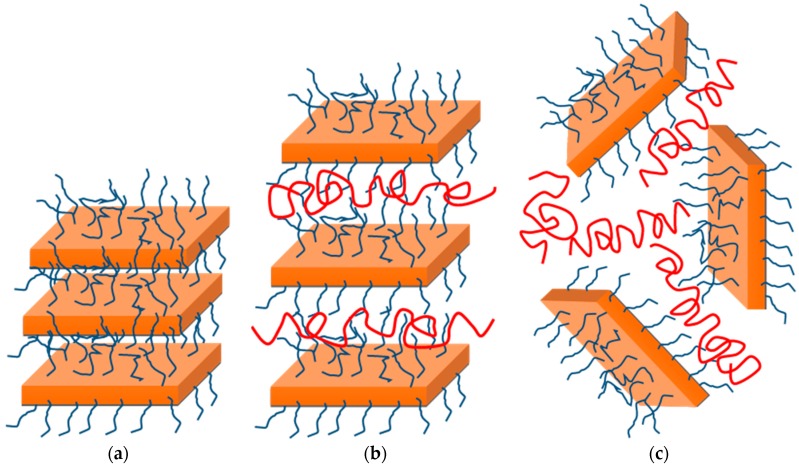
Schematic illustration of neat organically modified montmorillonite (OMMT) (**a**) and of intercalated (**b**) and exfoliated (**c**) structures of the PMMA/OMMT nanocomposites (PMMA macromolecules are denoted in red colour).

In our previous work, from both X-Ray Diffraction (XRD) analysis and TEM it has been suggested that PMMA nanocomposites containing 1 wt % Cloisite 25A, 1 wt % Cloisite 30B and 1 wt % AA-MMT are mainly exfoliated systems [15,38]. Moreover, from the XRD patterns it was concluded that nanocomposites with 1 wt % Cloisite 15A seem to be partially exfoliated, while for clay loadings higher than 1 wt % ΟΜΜΤ partially exfoliated and intercalated structures were revealed [15]. The intercalated and exfoliated structures of the PMMA/OMMT nanocomposites together with a schematic illustration of neat OMMT appear in Scheme 1. Figure 1a,b show indicative TEM photos of the PMMA/OMMT nanocomposites with 1 and 2 wt % Cloisite 15A respectively, where organic phase is depicted as a bright area while clay nanoparticles occur as dark narrow bands. In each case, it is obvious that discrete clay layers may co-exist with two and three layer clusters into the polymer matrix. This implies that there is a part of organomodified MMT nanoparticles retaining its intercalated characteristic structure and thus yielding a partially exfoliated structure.

### 4.2. Kinetics of Thermal Degradation

#### 4.2.1. Effect of Heating Rate

**Figure 2:**
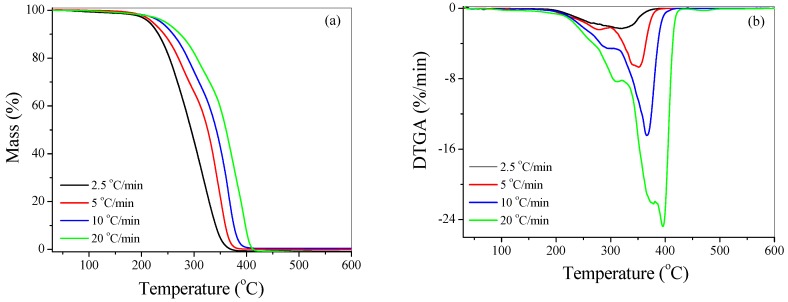
The heating rate efficacy on the thermogravimetric analysis (TGA) scans (**a**) and differential TGA (DTGA) curves (**b**) of neat PMMA.

**Figure 3:**
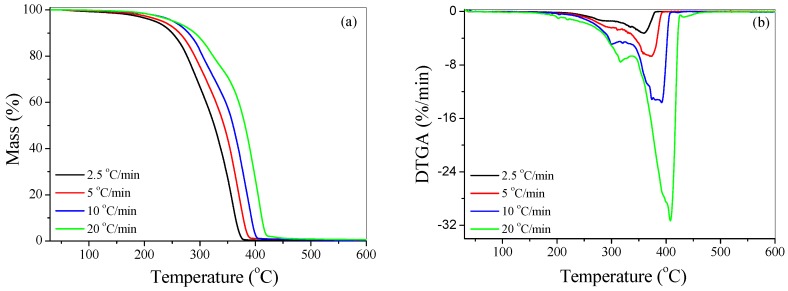
The heating rate efficacy on the TGA scans (**a**) and DTGA curves (**b**) of PMMA + 1 wt % Cloisite 15A.

Figure 2a and Figure 3a show the TGA scans of neat PMMA and PMMA + 1 wt % Cloisite 15A nanocomposite correspondingly, at the following heating rates: 2.5, 5, 10 and 20 °C/min. The differential TGA (DTGA) plots of these materials obtained at the above heating rates are illustrated in Figure 2b and Figure 3b, respectively. In each case, it is obvious that an increment in the heating rate displace the TGA and DTGA curves to higher temperatures. This behaviour could be attributed to the slower response of the heated sample against higher heating rates and has been also referred by other researchers [49,50,51,52]. The increase of the mass loss rate observed in DTGA curves is caused by the decreased time interval of thermal decomposition due to the higher heating rate [49,52,53]. Furthermore, both, neat PMMA and PMMA + 1 wt % Cloisite 15A nanocomposite were found to degrade mostly through a two-step reaction regardless the change of heating rate. In particular, for pure PMMA the first step occurs mainly at 290 °C, while the second at 365 °C for heating rate 10 °C/min. According to Peterson et al. [49], thermal degradation of PMMA occurs in two steps at temperature ranges of 180–350 °C and 350–400 °C respectively, for heating rates over 3.7 °C/min. The first step is due to the degradation of thermally weaker bonds and terminal groups such as head to head or tail to tail bonds, vinyl-groups and initiator fragments sited in the polymer chain, while the second step can be attributed to the random scission and depropagation [54,55,56,57,58]. The temperatures where thermal degradation starts, that is, at 1% (T_1%_), as well as at 10% (T_10%_) and 50% (T_50%_) for each sample studied at different heating rates are provided at Table 1. In the same Table the temperatures where the first and the second degradation peak appears are also included. It was observed that increasing heating rates shifts all temperatures to higher values. In addition, the effect of the OMMT was more pronounced on the first degradation peak temperature, whereas a slight effect was observed in the second degradation peak where random scission of the main chain backbone occurs. It was thus clear that the presence of the nano-clay has a better stabilizing and protecting effect on the degradation of the thermally weaker bonds. The char residue was near 0 for neat PMMA, while higher in the presence of the OMMT.

#### 4.2.2. Isoconversional Kinetic Analysis

**Figure 4:**
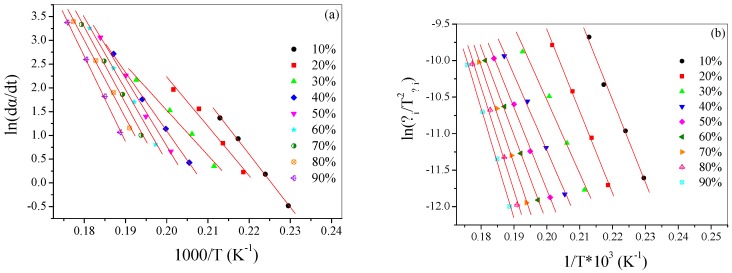
Representative plots of ln(d*α*/d*t*) vs. 1/*T* based on the differential isoconversional method of Friedman (**a**), and ln(*β_i_*/*T^2^_α__,i_*) vs. 1/*T* according to the integral isoconversional method of Coats and Redfern (**b**) for the PMMA + 1 wt % Cloisite 15A nanocomposite.

**Figure 5:**
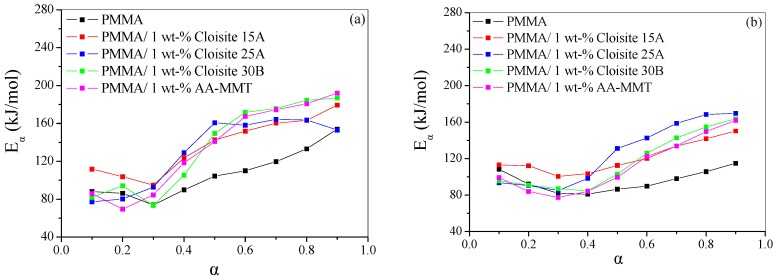
Effect of the OMMT type on the correlation between activation energy and extent of thermal degradation for PMMA nanocomposites determined using the method of Friedman (**a**); and the method of Coats and Redfern (**b**).

**Figure 6:**
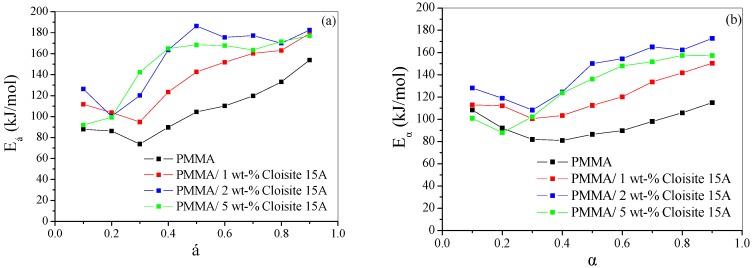
Effect of the OMMT clay content on the correlation between activation energy and the extent of thermal degradation for PMMA nanocomposites determined using the method of Friedman (**a**); and the method of Coats and Redfern (**b**).

In this part the TGA data obtained for pristine PMMA and its corresponding nanocomposites are subjected to isoconversional kinetic analysis, and the effective activation energy, *E_α_*, is determined as a function of the extent of thermal degradation, *α*. The differential method of Friedman was applied for the PMMA + 1 wt % Cloisite 15A nanocomposite leading in representative plots of ln(d*α*/d*t*) versus 1/*T* at different *α* values that appear in Figure 4a. It is clear that a satisfactory correlation of experimental data is presented in almost all series. The slope of these lines is used to determine the *E_α_* for pure PMMA and its nanocomposites. The plots illustrated in Figure 5a and Figure 6a represent the effectiveness of different type and amount of OMMT respectively on the *E_α_* value changes versus *α*. The slopes arisen from plots of ln(*β/T^2^*) versus 1/*T* at different *α*values according to the integral method of Coats-Redfern resulted in the calculation of the corresponding *E_α_*. Typical plots for PMMA + 1 wt % Cloisite 15A nanocomposite appear in Figure 4b and *E_α_* changes at different *α* values are also plotted in Figure 5b and Figure 6b.

Figure 5 and Figure 6 show higher *E_α_* values for all nanocomposites relative to pure PMMA at *α* values higher than 0.3. This observation confirms the higher thermal stability of nanocomposites compared to virgin PMMA that has been reported for the majority of them in our previous work [15,38]. In addition, the initial decrease of *E_α_* at low *α* values and the following gradual augmentation verify the existence of a two-step degradation mechanism in each case. This observation could be associated with the presence of weak bonds in the polymer structure that may lead to a somewhat easy commencement of thermal degradation [40]. Once the weak bond cleavage is completed, the degradation process is governed by a random scission generally demanding higher activation energy values [41].

Concerning the different type of OMMT (Figure 5a), *E_α_* starts from initial low value at *α*= 0.1 and can increase to a maximum of almost 192 kJ/mol at *α* = 0.9 for nanocomposite containing 1 wt % AA-MMT (according to the Friedman method). The exfoliated structure of the afore-mentioned nanocomposite could possibly account for this maximum *E_a_* value, indicating the best thermal stability at large conversion of degradation. On the basis of Coats-Redfern method, the same material exhibited a maximum *E_α_* value of 163 kJ/mol at *α* = 0.9. It is also clear that the presence of 1 wt % clay results in *E_α_* values that are at least 35 kJ/mol higher compared to pure PMMA for conversions above 0.5 (according to the Friedman method). The very high values of *E_α_* calculated at large extends of degradation from both methods explain the significant contribution of clay in the protection of nanocomposites against the thermal degradation.

In terms of the effect of the OMMT amount on the activation energy, from Figure 6a it is obvious that clay loading higher than 1 wt % leads to an increment of *E_α_* value at lower extent of degradation (over α = 0.2) compared with pure PMMA and its nanocomposite with 1 wt % OMMT (over α = 0.3) (according to the Friedman method), showing a better thermal stability at low conversions. According to both Friedman and Coats-Redfern methods, nanocomposite containing 2 wt % OMMT exhibited the highest *E_α_* values for α = 0.4–0.9, disclosing a better ability to withstand thermal degradation at the second step maybe due to its partially exfoliated structure. In particular, the maximum *E_α_* value is 186 kJ/mol and 173 kJ/mol at α = 0.5 for Friedman and Coats-Redfern method respectively. Further adding of clay nanoparticles up to 5 wt % does not seem to improve significantly the *E_α_* values for *α* = 0.4–0.9, as a possible presence of some tactoids in the intercalated structure could affect the thermal stability of the nanocomposite at the second step of degradation.

**Figure 7:**
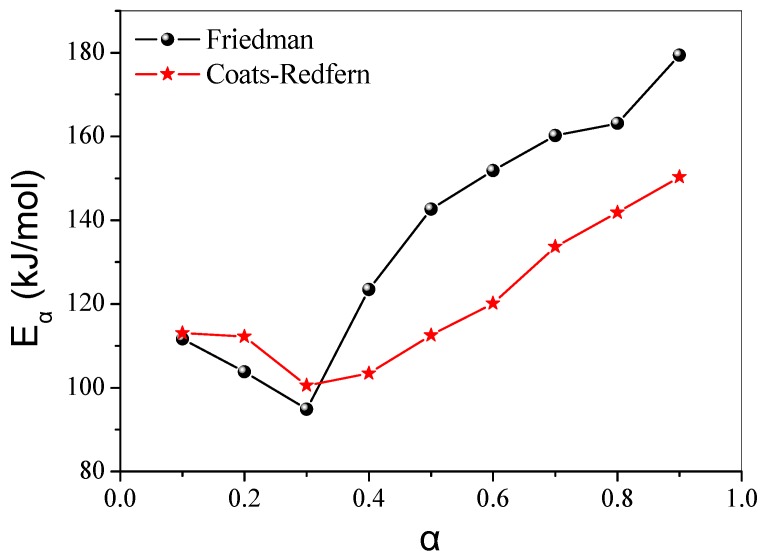
Correlation plots of effective activation energy versus the extent of degradation for PMMA + 1 wt % Cloisite 15A, determined using different methods.

From the data plotted in Figure 7 it is shown that both Friedman and Coats-Redfern methods are capable of foreseeing nearly a similar attitude of activation energy when correlated to the extent of thermal degradation for PMMA nanocomposite with 1 wt % Cloisite 15A. Systematic errors induced through integration method may be responsible for the differentiations observed regarding the *E_α_* values. Furthermore, the differential method of Friedman could be considered susceptible to experimental noise as it is based on immediate rate values, whereas mathematical approaches are taken account through integral Coats-Redfern method.

### 4.3. Dynamic Mechanical Thermal Analysis (DMTA)

**Figure 8:**
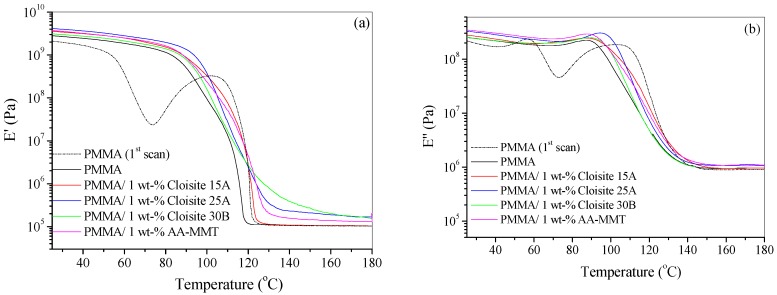
Storage modulus E′ (**a**), and loss modulus E″; (**b**) as a function of temperature of PMMA nanocomposites containing different OMMT types.

The viscoelastic properties of the pure PMMA and its nanocomposites were measured as a function of temperature by DMTA. The results obtained were based on the response of a given specimen to a cyclic deformation in function of the temperature at frequency 1 Hz. The dynamic mechanical properties for each material were determined again through a second scan under the same experimental conditions. Plots of the storage modulus, E′, and the loss modulus, E″, versus temperature for neat PMMA and its nanocomposites with different types of OMMT clay, according to the data of the second scan, are represented in Figure 8a,b. The same Figure also shows as indicative the first scan for pure PMMA. The peak detected for the first scan at ~73 °C is reasonably attributed to the relaxation of macromolecular chains which has been occurred during the molding process for the preparation of specimens. Similar behavior was also observed for all the corresponding nanocomposites. It is apparent that in the E′ curve (Figure 8a) the two regions where the E′ decreases abruptly for the first scan are replaced by a unique region throughout the second scan, that is, a glass transition region occurs. Moreover, the E″ shows a maximum peak in the second scan for the same reason (Figure 8b).

Concerning the different type of OMMT clay (Figure 8), the curves Ε′ and E″ were shifted to higher values for all nanocomposites in comparison to the virgin PMMA, but no clear tendency was detected in the viscoelastic performance of the nanocomposites at all the temperature range that was studied. According to the data of Table 2, an augmentation of E′ for the nanocomposites with different types of Cloisite at 25 °C was observed. More specifically, the nanocomposite containing 1 wt % Cloisite 25A and AA-MMT exhibited an increase in E′ of 47 and 32% respectively, showing their improved stiffness compared with the pure PMMA. This behaviour can be ascribed to the restricted segmental motions at the organic–inorganic interface: because of the large aspect ratio of the nanometric silicate layers, the polymer chains are well confined in the interlayer galleries at the nanoscale level [33].

**Figure 9:**
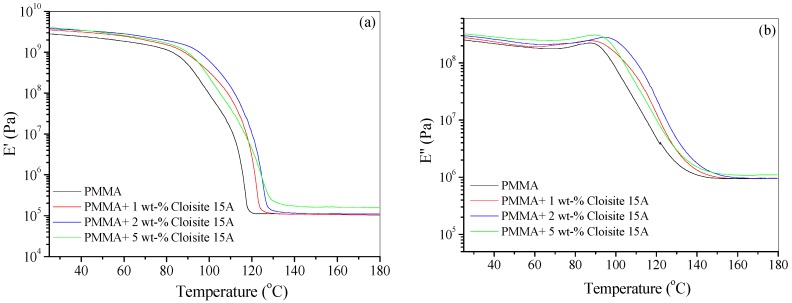

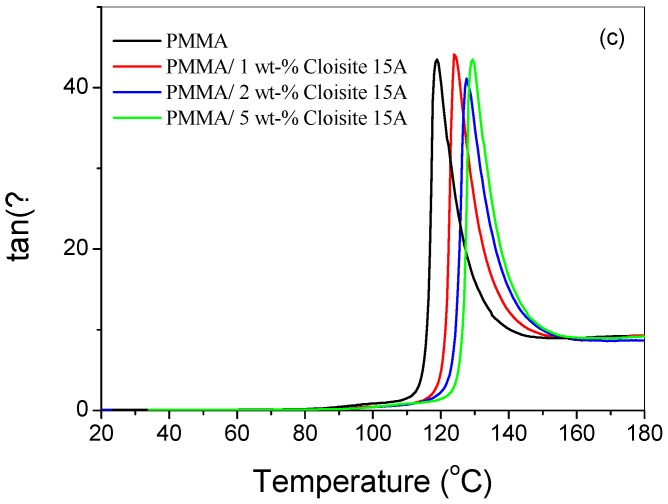
Storage modulus E′ (**a**), loss modulus E″ (**b**) and loss tangent tan(δ) (**c**) as a function of temperature of PMMA nanocomposites with different amount of OMMT clay.

**Figure 10:**
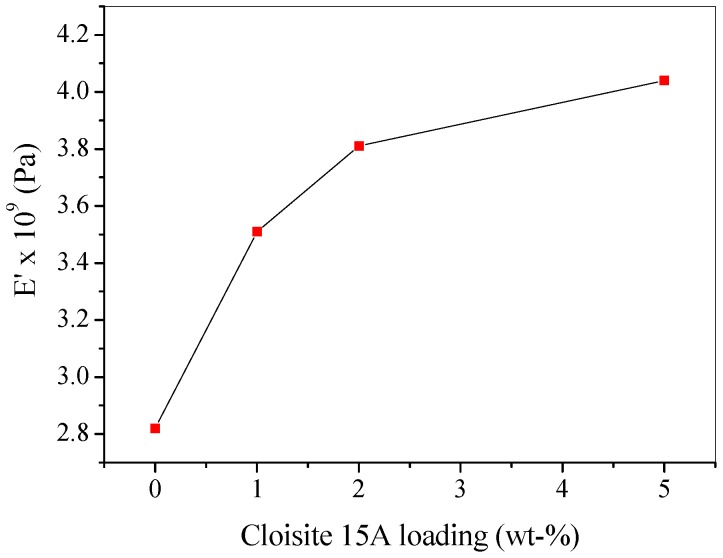
Effect of clay loading on the storage modulus E′ of PMMA nanocomposites at 25 °C.

Figure 9 reveals that an increase of the amount of OMMT clay up to 2 wt % results in a gradual shift of E′ and E″ curves to higher values. Further addition of clay rather seems to decrease the values of the above parameters. In addition, Figure 10 illustrates the effect of clay loading on the E′ of PMMA nanocomposites at 25 °C. It is obvious that by increasing the relative amount of Cloisite 15A up to 5 wt % the stiffness of the nanocomposite improves gradually, that is, an increment in E′ of 43%. Liaw et al. [59] reported a relatively small increase of stiffness due to clay addition at room temperature for PMMA nanocomposites with Cloisite 15A, prepared by twin-screw compounding method. When 9 wt % clay was added, the PMMA/clay system showed an increase up to 33% in the storage modulus. In addition, the effect of the amount of OMMT on the variation of loss tangent (tan(δ)) with temperature appears in Figure 9c. As the amount of clay increases, the curves are shifted to higher temperatures. From the peak point of these curves the glass transition temperature of the polymer can be estimated. The values thus measured were 119, 124, 127.5 and 129.4 for neat PMMA and the nanocomposites with 1, 2 and 5 wt % of OMMT, respectively. Therefore, it seems that the addition of the clay also increases the Tg of the polymer. It should be pointed here that the Tg values measured by DMTA are always higher than the corresponding estimated by DSC since they are based on a different way of measurement.

## 5. Conclusions

In this investigation, PMMA/OMMT clay nanocomposites were successfully prepared by in situ bulk polymerization and their thermal degradation kinetics were studied using TGA. Both pure PMMA and its nanocomposites were found to degrade through a two-step process. An isoconversional kinetic analysis of the obtained TGA results confirmed the aforementioned way of thermal degradation. An improvement of *E_α_* value for all nanocomposites in comparison to pure PMMA at *α* values higher than 0.3 was observed, indicating their higher thermal stability, while nanocomposite with 2 wt % OMMT clay exhibited the highest *E_α_* values for α over than 0.4. The viscoelastic properties of the above nanocomposites were also measured by means of DMTA, showing a gradual improvement of stiffness for clay loading up to 5 wt % at room temperature. The enhanced thermal degradation and viscoelastic properties of these PMMA/OMMT nanocomposites make them excellent candidates in applications requiring high thermal stability and mechanical strength, such as in dental materials as dentures, car lights, or in packaging.

## Figures and Tables

**Table 1:** Temperatures where 1% (T_1%_), 10% (T_10%_) and 50% (T_50%_) thermal degradation occurs, as well as of the first and second degradation peak and residue for each sample studied at different heating rates.

Sample	Heating Rate (°C/min)	T_1%_ (°C)	T_10%_ (°C)	T_50%_ (°C)	T_p1_ (°C)	T_p2_ (°C)	Residue (^%^)
PMMA	2.5	136	231	294	271	319	0
	5	143	242	326	277	348	0
	10	154	259	342	293	366	0.1
	20	162	272	360	311	392	0.3
PMMA + 1 wt % Cloisite 15A	2.5	142	248	326	326	326	0.1
	5	154	262	344	344	346	0.3
	10	176	279	360	360	364	0.6
	20	183	290	381	381	387	0.8

**Table 2:** Storage modulus E′ of PMMA nanocomposites with different types of OMMT clay at 25 °C.

Sample	Storage Modulus, E′ × 10^9^ (Pa)
PMMA	2.82
PMMA + 1 wt % Cloisite 15A	3.54
PMMA + 1 wt % Cloisite 25A	4.14
PMMA + 1 wt % Cloisite 30B	3.11
PMMA + 1 wt % AA-MMT	3.71
